# Permissive and restricted virus infection of murine embryonic stem cells

**DOI:** 10.1099/vir.0.043406-0

**Published:** 2012-10

**Authors:** Rachael Wash, Sabrina Calabressi, Stephanie Franz, Samantha J. Griffiths, David Goulding, E-Pien Tan, Helen Wise, Paul Digard, Jürgen Haas, Stacey Efstathiou, Paul Kellam

**Affiliations:** 1 Wellcome Trust Sanger Institute, Wellcome Trust Genome Campus, Hinxton, Cambridge, CB10 1SA, UK; 2 Division of Pathway Medicine, The University of Edinburgh, Old College, South Bridge, Edinburgh, EH8 9YL, UK; 3 Department of Pathology, University of Cambridge, Tennis Court Road, Cambridge, CB2 1QP, UK; 4 UCL/MRC Centre for Medical Molecular Virology, Department of Infection, University College London, London WC1E 6BT, UK

## Abstract

Recent RNA interference (RNAi) studies have identified many host proteins that modulate virus infection, but small interfering RNA ‘off-target’ effects and the use of transformed cell lines limit their conclusiveness. As murine embryonic stem (mES) cells can be genetically modified and resources exist where many and eventually all known mouse genes are insertionally inactivated, it was reasoned that mES cells would provide a useful alternative to RNAi screens. Beyond allowing investigation of host–pathogen interactions *in vitro*, mES cells have the potential to differentiate into other primary cell types, as well as being used to generate knockout mice for *in vivo* studies. However, mES cells are poorly characterized for virus infection. To investigate whether ES cells can be used to explore host–virus interactions, this study characterized the responses of mES cells following infection by herpes simplex virus type 1 (HSV-1) and influenza A virus. HSV-1 replicated lytically in mES cells, although mES cells were less permissive than most other cell types tested. Influenza virus was able to enter mES cells and express some viral proteins, but the replication cycle was incomplete and no infectious virus was produced. Knockdown of the host protein AHCYL1 in mES cells reduced HSV-1 replication, showing the potential for using mES cells to study host–virus interactions. Transcriptional profiling, however, indicated the lack of an efficient innate immune response in these cells. mES cells may thus be useful to identify host proteins that play a role in virus replication, but they are not suitable to determine factors that are involved in innate host defence.

## Introduction

Viruses are obligate intracellular parasites that hijack the host’s cellular machinery during their replication cycle. RNA interference (RNAi) screens have proved a very powerful tool for reducing expression of host genes during infection with many different viruses, including influenza virus and human immunodeficiency virus, thereby identifying host proteins that affect virus replication. Such host genes can be grouped into virus replication dependence factors (VRDFs), those host proteins that are required for virus replication, and virus restriction factors (VRFs), those host proteins that block virus infection (Brass *et al.*, 2008; Karlas *et al.*, 2010; König *et al.*, 2010; Krishnan *et al.*, 2008; Zhou *et al.*, 2008). However, problems with RNAi methods can give ‘off-target’ effects or incomplete knockdown. Furthermore, variation between RNAi studies examining the same virus can be influenced by the cell line, virus strain and methodology, resulting in a lack of overlap between the RNAi gene sets identified (Bartz & Jackson, 2005; Watanabe *et al.*, 2010; Zhou *et al.*, 2008). The use of classical genetic screens, through insertional inactivation in haploid cells, has recently identified VRDFs to influenza virus; however, the unusual karyotype of chronic lymphoblastic leukaemia may limit this approach (Carette *et al.*, 2009).

Ultimately, the functional validation of RNAi results requires either the identification of corresponding gene defects in the authentic host or the production of knockout (KO) mice and assessment of virus infection. Importantly, comparison of murine and human genomes has shown that there is a homologue for ~99 % of murine genes in the human genome (Chinwalla *et al.*, 2002). An international programme where KOs for all genes of the mouse are being produced is under way (Austin *et al.*, 2004), as well as infection challenge studies in different mouse strains (Boon *et al.*, 2011), and this is now supported by the complete genome sequences of 17 mouse strains (Keane *et al.*, 2011; Yalcin *et al.*, 2011). In addition, the four members of the KO mouse consortium, namely the European Conditional Mouse Mutagenesis Programme (EUCOMM; http://www.knockoutmouse.org/about/eucomm), the Knockout Mouse Programme (http://www.komp.org), the Canadian North American Conditional Mouse Mutagenesis Project (http://www.norcomm.org) and the Texas Institute for Genomic Research (http://www.tigm.org), have produced 10 000 heterozygous KO murine embryonic stem (mES) cells and hundreds of homozygous KO mice, many of which are of interest because the genes have been identified previously as VRFs or VRDFs in viral screens. Furthermore, recent advances in gene targeting strategies have also resulted in the ability to produce inducible homozygous mutant mES cells (Li *et al.*, 2010; Tate & Skarnes, 2011). This raises the possibility of using murine KO mES cells both for random library screening and for confirmation experiments before deriving KO mice. mES cells are derived from the inner cell mass of blastocysts, and are self-renewing and remain pluripotent if cultured under the correct conditions. They can also be differentiated into many cell types, such as neuronal, dendritic and hepatic cell lineages (Bibel *et al.*, 2004; Fairchild *et al.*, 2000; Soto-Gutiérrez *et al.*, 2007), offering great potential for investigating viruses with specific cell tropisms.

Previous work has demonstrated that mES cells can be used for studying cellular interactions with bacterial pathogens. After exposure of mES cells to *Salmonella enterica*, *Shigella flexneri* or *Escherichia coli*, the bacteria were detected in intracellular locations similar to those observed for differentiated cells. In comparison with wild-type mES cells, infection of mES cells defective in a gene required for cholesterol biosynthesis resulted in lower levels of bacterial replication, showing the potential of using KO mES cells for looking at bacteria–host interactions (Yu *et al.*, 2009). In addition, it has been demonstrated that human ES (hES) cells are susceptible to coxsackievirus B and produce viable virus particles (Scassa *et al.*, 2011). However, apart from work with retroviral vectors (Wang & Bradley, 2007), less is known about the ability of human viruses to productively infect and replicate in mES cells. Here, we characterized virus infection of mES cells using influenza A virus and herpes simplex virus type 1 (HSV-1). These viruses were selected because they are important human pathogens (Deshpande *et al.*, 2000; Schoenbaum, 2001) for which RNAi screens have been carried out (Brass *et al.*, 2009; Karlas *et al.*, 2010), they are known to infect other murine cell lines (Bolovan *et al.*, 1994; Shinya *et al.*, 2004) and they have well-defined mouse models (Matsuoka *et al.*, 2009; Mester & Rouse, 1991).

We showed that mES cells are permissive for HSV-1 but restrict the full influenza virus replication cycle. KO of an HSV-1 dependency factor in mES cells resulted in attenuated HSV-1 replication, demonstrating the use of mES cells as a genetic resource for host–virus interactions. However, the lack of innate immune responses following infection suggests that mES cells are not suitable for determining factors that are involved in innate host defence.

## Results

### Entry of HSV-1 and influenza into mES cells

**Fig. 1.**
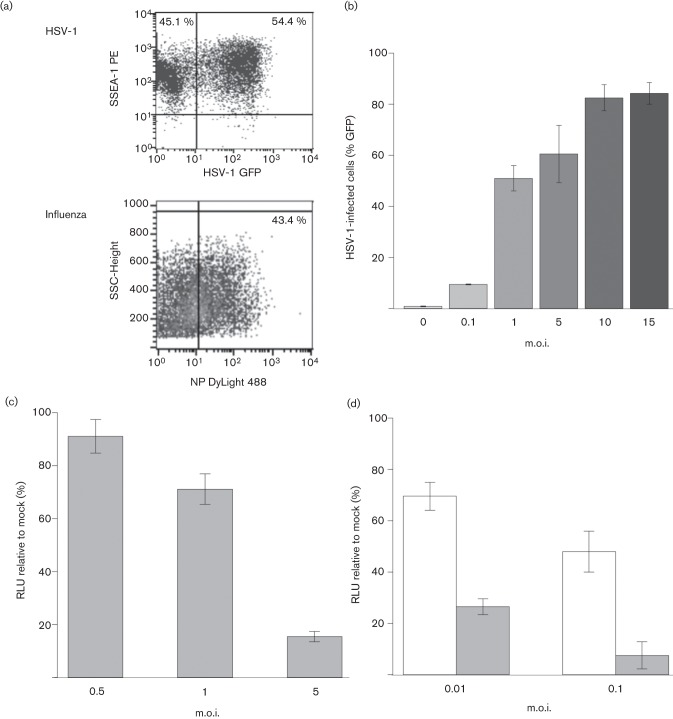
(a) Flow cytometric detection of stage-specific embryonic antigen-1 (SSEA-1) expression in mES cells at 24 h after infection with HSV-1 (GFP) at an m.o.i. of 1 (upper panel) or nucleoprotein (NP) expression in mES cells at 24 h after infection with influenza virus A/WSN/33 at m.o.i. of 0.1 (lower panel). (b) HSV-1 infection at different m.o.i. assessed by flow cytometric detection of GFP expression from HSV-1 in mES cells. Samples were taken in triplicate and analysed at 24 h p.i. Results are shown as means±sd. (c) mES cell viability after HSV-1 infection. Cells were infected in suspension with HSV-1 at an m.o.i. of 0.5, 1 and 5. Viability was assessed at 48 h p.i. using a CellTiter-Glo kit. A significant decrease was seen (*P*≤0.001) for samples infected at m.o.i. of 1 and 5 when compared with mock-infected samples (Student’s *t*-test). (d) mES cell viability after A/WSN/33 infection. Cells were infected in suspension with A/WSN/33 at an m.o.i. of 0.01 or 0.1. Viability was assessed at 24 (open bars) and 48 (shaded bars) h p.i. A significant decrease was seen (*P*≤0.0002) for both treatments when compared with mock-infected samples (Student’s *t*-test).

We assessed the ability of HSV-1 and influenza virus A/WSN/33 to infect the wild-type mES cell line JM8A1.N3. HSV-1 infection was detected through GFP expression under the control of the cytomegalovirus (CMV) immediate-early 1 (IE1) promoter in HSV-1 C12 (Fig. 1a). The stem cell phenotype of JM8A1.N3 was maintained during infection, as determined by the maintenance of expression of SSEA-1, a surface antigen that is a marker for undifferentiated murine stem cells (Fig. 1a). Similarly, entry and initiation of viral gene expression by influenza virus was confirmed by detection of NP expression at 24 h post-infection (p.i.) in mES cells (Fig. 1a). HSV-1 infection of JM8A1.N3 cells increased with higher m.o.i., with an m.o.i. of 10 resulting in 80 % of the mES cells expressing virus-driven GFP by 24 h p.i. (Fig. 1b).

Following infection of mES cells with HSV-1 or A/WSN/33, a cytopathic effect was observed by 24 h p.i., with cells rounding up and detaching from the culture vessels. For HSV-1-infected cells, viability remained similar to that of mock-infected cells at 24 h p.i. (data not shown) but decreased significantly as assessed by cellular ATP levels by 48 h p.i. at an m.o.i. of 1 and 5 (Fig. 1c, *P*≤0.001). Infection with influenza also caused a loss in cell viability, even at lower m.o.i. and as early as 24 h p.i. (Fig. 1d, *P*≤0.0002).

### Viral protein production in mES cells

**Fig. 2.**
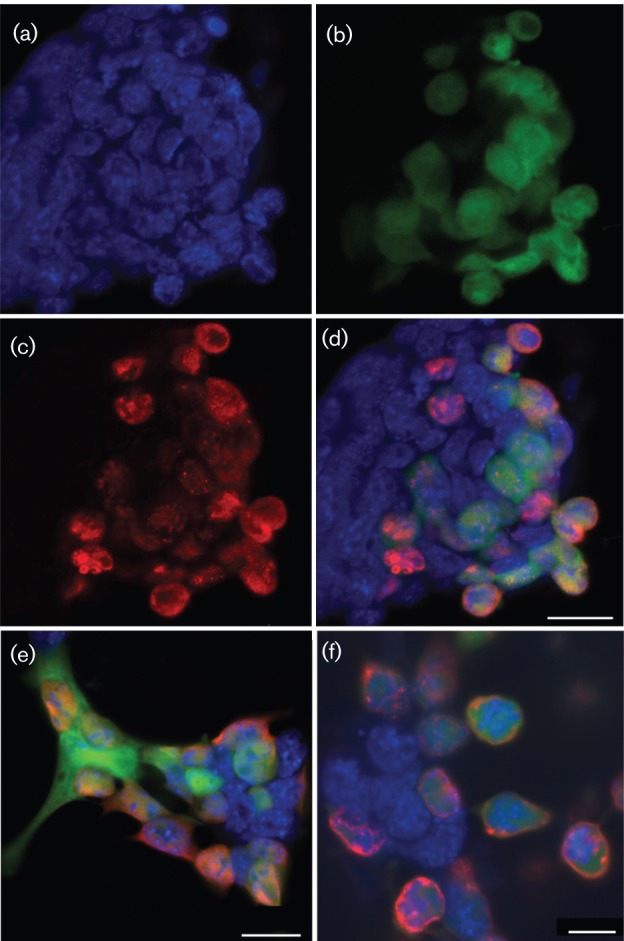
Confocal microscopy of HSV-1-infected mES cells at 24 h p.i. GFP expression from HSV-1 infection was visible as green fluorescence, HSV-1 gene-specific antibody staining was shown by red fluorescence and mES cell nuclei were stained with DAPI (blue). (a) DAPI-stained mES cell nuclei. (b) GFP-expressing HSV-1-infected mES cells. (c) HSV-1 ICP8 staining with DyLight 549. (d) Merged image of (a), (b) and (c). (e) Merged image for HSV-1-infected mES cells with HSV-1 ICP27 staining. (f) Merged image for HSV-1-infected mES cells with HSV-1 glycoprotein C staining. Bars, 20 µm (d, e); 10 µm (f).

**Fig. 3.**
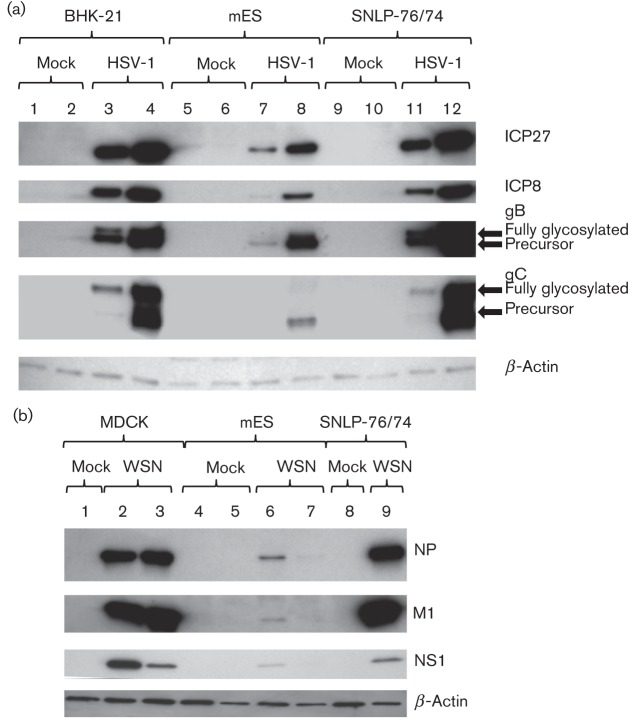
(a) Western blot analysis of cells infected with HSV-1 C12. BHK-21, mES or SNLP-76/7-4 cells were infected at an m.o.i. of 1 and cell lysates were collected at 6 (lanes 1, 3, 5, 7, 9 and 11) or 24 (lanes 2, 4, 6, 8, 10 and 12) h p.i. Membranes were probed with HSV-1-specific antibodies against ICP27, ICP8, gB and gC, followed by secondary antibody conjugated to HRP. (b) Western blot analysis of cells infected with A/WSN/33 (WSN). MDCK, mES or SNLP-76/7-4 cells were infected at an m.o.i. of 1 and cell lysates were collected at 10 (lanes 2, 4 and 6) or 24 (lanes 1, 3, 5 and 7–9) h p.i. Membranes were probed with influenza-specific antibodies, followed by secondary antibody conjugated to HRP. Antibody against β-actin was used as a loading control in both experiments.

Uninfected mES cells grow in discrete colonies, rather than monolayers, with the cells having large nuclei and minimal cytoplasm (Fig. 2a). Following infection of mES cells, we examined the extent of HSV-1 gene expression using antibodies against HSV-1 ICP27, ICP8 and glycoprotein C (gC), which are immediate-early (IE), early (E) and late (L) gene products, respectively, using immunofluorescent microscopy. The majority of cells expressing virus-encoded GFP also co-expressed the HSV-1 IE, E and L viral proteins (Fig. 2b). As expected, expression of ICP8 and ICP27 were observed in the nuclei of infected cells (Fig. 2c–e), whilst gC was expressed predominantly on the cell surface (Fig. 2f). mES cells infected at the same m.o.i. as baby hamster kidney (BHK-21) and murine embryonic fibroblast feeder (SNLP 76/7-4) cells expressed less viral protein at 6 and 24 h p.i. (Fig. 3a). HSV-1 gB and gC glycosylation was defective in mES cells. Precursor gB and gC proteins were clearly expressed by 24 h; however, glycosylated high-molecular-mass gB and gC proteins were not detected (Fig. 3a). For influenza virus infection, viral protein expression in mES cells was severely reduced in comparison with fully permissive Madin–Derby canine kidney (MDCK) or SNLP 76/7-4 cells. Although NP, M1 and NS1 expression was observed at 10 h p.i., no expression was detectable by 24 h by Western blotting (Fig. 3b).

### Replication of HSV-1 and influenza virus in mES cells

**Fig. 4.**
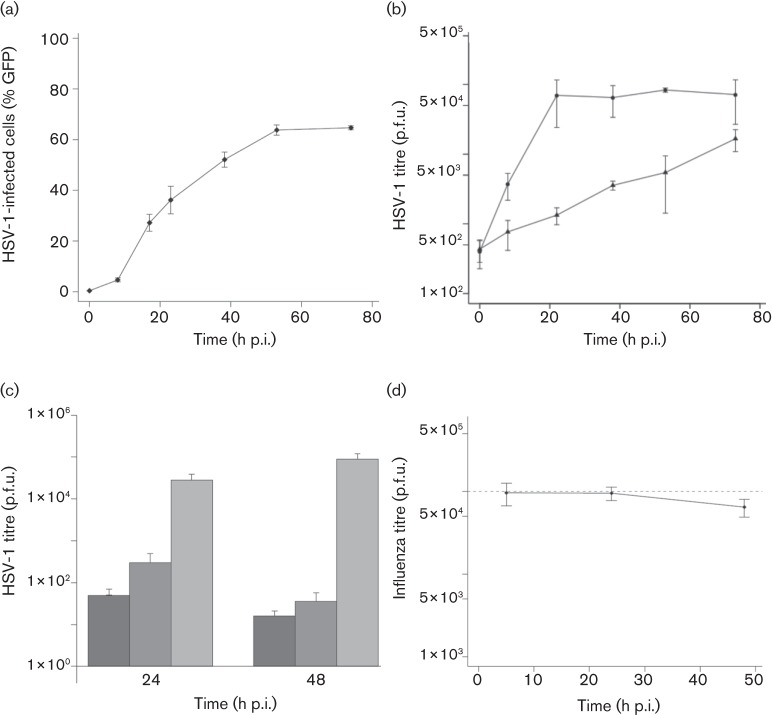
(a) HSV-1 infection kinetics in mES cells infected at an m.o.i. of 1. Samples were taken from 0 to 76 h p.i. and GFP expression by infected cells was analysed by flow cytometry. (b) Generation of productive virus was also quantified by plaque assays of infected mES cell lysates (•) and culture medium (▴) on BHK-21 cells. (c) Inhibition of HSV-1 replication (m.o.i. of 1) in mES cells using 44.4 µM ACV or 0.2 µM FIAU. Cell lysates were harvested at 24 and 48 h p.i. and the generation of productive virus was quantified by plaque assay on BHK-21 cells. ACV, Dark grey-shaded columns; FIAU, mid-grey-shaded columns; no drug, light grey-shaded columns. (d) A/WSN/33 infection kinetics in mES cells infected in suspension at an m.o.i. of 0.2. Samples of culture medium were taken at 6, 24 and 48 h p.i. and generation of productive virus was quantified by plaque assay on MDCK cells. Input virus is indicated by a dashed line.

We determined whether HSV-1 could form spreading infections in mES cell by flow cytometric analysis of infected cells. For HSV-1 infection at an m.o.i. of 1 (based on the titre of the virus in BHK-21 cells), GFP expression increased over the 53 h following infection, reaching a maximum of 63 % of cells expressing GFP by this time (Fig. 4a). The majority of the virus was cell associated (Fig. 4b). We confirmed full virus lytic replication and spreading infection by inhibiting HSV-1 replication with acyclovir (ACV) or 1-(2′deoxy-2′-fluoro-1-β-d-arabinofuranosyl)-5-iodo-uracil (FIAU), with both virus replication inhibitors significantly reducing HSV-1 replication (Fig. 4c). Electron micrographs of HSV-1-infected mES cells (Figs S1 and S2, available in JGV Online) revealed HSV-1 intracellular particles in nuclear virus factories, associated with the nuclear membrane and at the cell surface. We assessed the spread of influenza virus infection both in mES cells growing as clumps and by infecting disrupted mES cells in suspension. As this was performed in the presence of serum needed to maintain the mES cells in their undifferentiated state, a trypsin-independent strain of influenza virus (A/WSN/33) (Goto & Kawaoka, 1998) was used. Irrespective of mES cell culture type, influenza virus failed to establish a spreading infection (Fig. 4d). We therefore concluded that mES cells support HSV-1 replication but not influenza A virus replication.

### Host response to infection

**Fig. 5.**
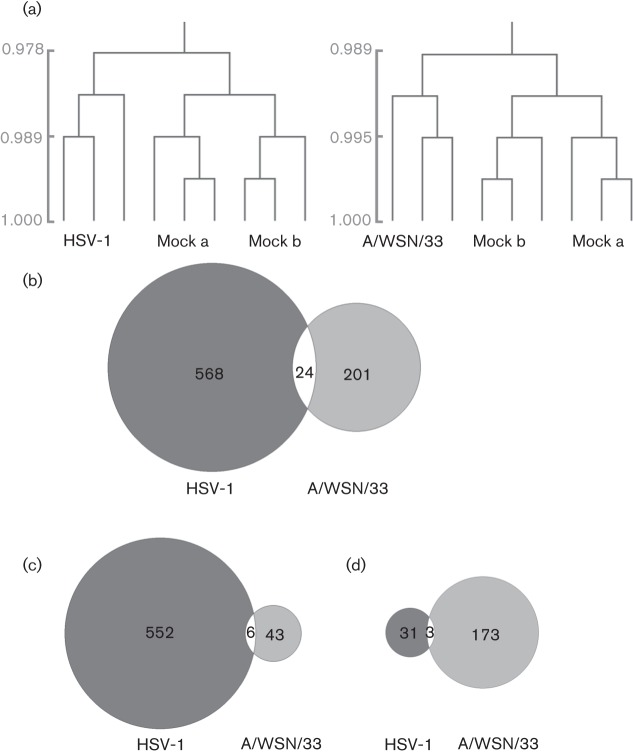
Microarray analysis of mES cells infected with HSV-1 or A/WSN/33, at 6 h p.i. (a) Hierarchical clustering of mock-infected samples compared with HSV-1- or A/WSN/33-infected samples. Infections were set up in triplicate and mock samples were taken from two independent experiments. The scale bars show Pearson correlation coefficients. (b) Venn diagrams of all significantly differentially expressed genes in mES cells infected with HSV-1 or A/WSN/33. Data were analysed using Significance Analysis for Microarrays (δ value of 1.5, false discovery rate <0.025, fold change ≥1.5). (c) Comparison of significantly upregulated genes only. (d) Comparison of significantly downregulated genes only.

mES cells are reported to be defective in an interferon response when infected by bacteria. Virus infection is a powerful inducer of type I interferons, as many virus components are recognized by pattern recognition receptors. We therefore used gene expression profiling of HSV-1- and influenza virus-infected mES cells at an early time point after infection to investigate whether innate immune responses were induced in mES cells. Hierarchal clustering analysis demonstrated that the expression profiles of HSV-1- and influenza virus A/WSN/33-infected mES cell samples clustered in groups distinct from mock-infected mES cells (Fig. 5a). Following infection, >200 genes were expressed differentially in virus-infected mES cells (Fig. 5b). Although there was some overlap between the host genes affected by HSV-1 and A/WSN/33, they were not necessarily regulated in the same direction. Whilst the majority of changes following HSV-1 infection were due to upregulation of genes, in A/WSN/33-infected mES cells a different pattern was observed and more genes were downregulated (Fig. 5c, d). Functional analysis of genes that exhibited significant changes showed that HSV-1 infection induced the upregulation of transcriptional processes, whereas infection with A/WSN/33 induced downregulation of transcriptional processes (Table 1). No downregulated genes were found to be functionally enriched in HSV-1-infected cells or upregulated genes for influenza virus-infected cells. Use of the interferome database identified five upregulated genes for HSV-1 and nine for A/WSN/33 that are involved in the type I interferon response (Table 2).

**Table 1.** Enrichment analysis (david) of significantly expressed genes in HSV-1- and influenza virus A/WSN/33-infected mES cells at 6 h p.i.

Biological process	No. genes	Total genes (%)	Enrichment score	*P* value
HSV-1-upregulated genes				
Regulation of transcription	49	8.78	14.26	1.3×10^−38^
Regulation of transcription from RNA polymerase II promoter	38	6.81	10.33	2.30×10^−41^
Transcription	26	4.66	3.51	6.40×10^−14^
Nucleosome assembly	5	0.9	3.12	7.70×10^−10^
Protein amino acid dephosphorylation	6	1.08	2.5	3.80×10^−11^
Nuclear division	8	1.43	2.08	7.40×10^−13^
A/WSN/33-downregulated genes				
Regulation of transcription	15	8.52	2.36	1.20×10^−10^

**Table 2.** Type I interferon-regulated genes, from the interferome database, that were significantly upregulated in HSV-1- or influenza virus A/WSN/33-infected mES cells at 6 h p.i.

Gene	Description	Fold change
HSV-1-upregulated genes		
Tbx3	T-box 3	1.55
Trex1	Three prime repair exonuclease 1	1.58
Tuba1c	Tubulin-α1C	1.82
Wdr43	WD repeat domain 43	1.61
Zfp36	Zinc finger protein 36	3.46
A/WSN/33-upregulated genes		
Chka	Choline kinase α	1.85
Eif2s2	Eukaryotic translation initiation factor 2, subunit 2 (β)	1.53
H2afj	H2A histone family, member J	1.52
Ifngr2	Interferon-γ receptor 2	2.21
Lgals4	Lectin, galactose binding, soluble 4	4.34
Prf1	Perforin 1 (pore forming protein)	2.78
Rbbp4	Retinoblastoma binding protein 4	1.51
Traf4	Tumour necrosis factor receptor associated factor 4	1.66
Ube2n	Ubiquitin-conjugating enzyme E2N	2.24

### Knockdown of host genes and virus infection

**Fig. 6.**
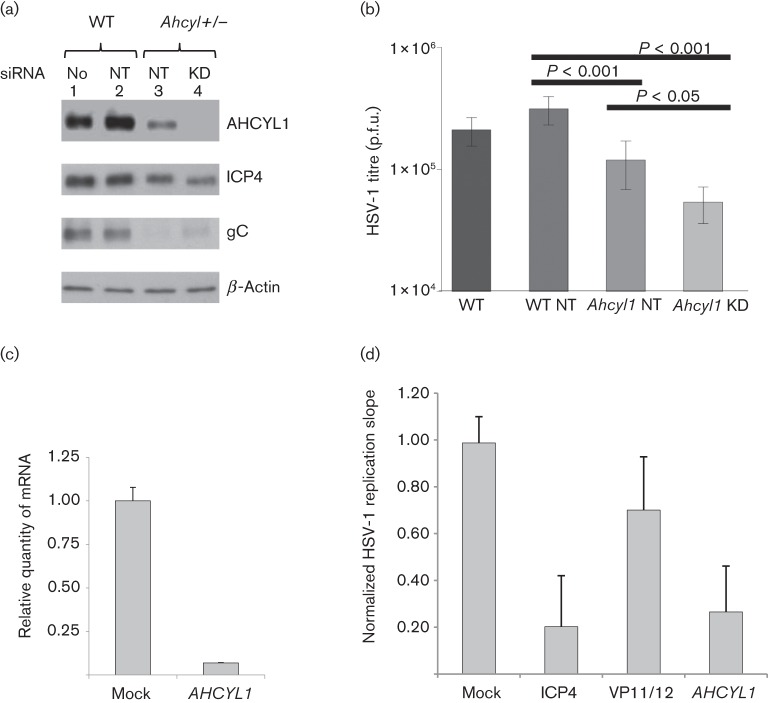
(a) Protein extracts from mES cells were analysed by Western blotting after treatment with siRNA for 48 h followed by HSV-1 infection (m.o.i. of 0.5). Membranes were probed with antibodies specific for AHCYL1, HSV-1 ICP4 (6 h p.i.) and HSV-1 gC (24 h p.i.). Antibody against β-actin was used as a loading control. (b) Generation of productive HSV-1 at 24 h p.i. in mES cells transfected with siRNA 48 h prior to infection. Student’s *t*-test was performed. WT, Wild-type mES cells; *Ahcyl^+/−^*, mES cells with heterozygous knockout for *Ahcyl1*; No, no RNAi; NT, non-targeting siRNA pool; KD, knockdown with *Ahcyl1*-specific siRNA pool. (c) siRNA depletion reduces mRNA expression levels of *AHCYL1* in HeLa cells. mRNA expression levels of *AHCYL1* at 48 h post-transfection were quantified by Taqman qPCR, normalized to the housekeeping gene hypoxanthine phosphoribosyltransferase 1 (*HPRT1*) and calibrated against mock-transfected cells. Mean gene knockdown is presented, with error bars representing sd of technical duplicates. (d) RNAi perturbation screen by kinetic analysis of HSV-1 replication. HeLa cells were reverse transfected with siRNA SMARTpools (four siRNAs per gene). After 48 h, the siRNAs were tested for cytotoxicity (three replicates) or the capacity to influence replication of the HSV-1 GFP reporter virus C12 (six replicates) from 24 to 80 h p.i. Replication was normalized to mock-transfected cells and compared with replication following knockdown of essential (ICP4, VP16) or non-essential (VP11/12) viral genes, or control RISC-free siRNA (not shown). HSV replication is presented as the normalized replication slope and is the mean±sd of six individual assay points.

We investigated whether mES cells knocked out for a gene identified in RNAi screens (Karlas *et al.*, 2010) and shown previously to be necessary to support HSV-1 replication (S. J. Griffiths, M. Koegl, C. Boutell, H. L. Zenner, C. M. Crump, O. Gonzalez, C. C. Friedel, G. Barry, K. Martin, M. H. Craigon, R. Chen, L. N. Kaza, E. Fossum, J. K. Fazakerley, S. Efstathiou, R. Zimmer, P. Ghazal and J. Haas, unpublished data) produced an analogous phenotype. Heterozygote KO mES cells for *Ahcyl1* (*Ahcyl1^+/−^*) expressed reduced levels of AHCYL1 protein (Fig. 6a, lane 3) relative to wild-type mES cells (Fig. 6a, lanes 1 and 2). *Ahcyl1^+/−^* cells still supported HSV-1 replication but with reduced HSV-1 ICP4 and gC expression (Fig. 6a, lane 3), as well as a significantly reduced output viral titre (Fig. 6b; *P*<0.001). Further knockdown of *Ahcyl1* using an RNAi pool resulted in undetectable levels of the protein but did not further reduce ICP4 and gC expression following HSV-1 infection (Fig. 6a, lane 4). However, the loss of detectable AHCYL1 expression led to a further significant reduction in the titre of replicated virus (Fig. 6b; *P*<0.05).

To confirm the requirement of AHCYL1 for HSV-1 replication, we used small interfering RNA (siRNA) knockdown of AHCYL1 in a human cell line. Following transfection, siRNA depletion of *AHCYL1* mRNA levels was confirmed by quantitative PCR (qPCR) (Fig. 6c). Replication of HSV-1 in HeLa cells transfected with *AHCYL1*, RNA-induced silencing complex (RISC)-free (negative control), ICP4 (essential HSV-1 gene) or VP11/12 (non-essential HSV-1 gene) siRNAs was compared, with replication being normalized to that of mock-transfected cells (Fig. 6d). When either the essential HSV-1 ICP4 gene or *AHCYL1* was knocked down, reductions of more than threefold in HSV-1 replication were observed.

## Discussion

Here, we showed that murine embryonic stem cells support infection with HSV-1 and influenza virus. HSV-1 was able to complete its replication cycle in mES cells, although they were less permissive than other cell lines. HSV-1 is able to replicate efficiently in non-human cells; however, the observed delayed expression of gC in mES cells and the expression of precursor but not glycosylated gC and gB proteins may contribute to reduced replication (Wenske & Courtney, 1983). HSV-1 gC has an important role in adsorption of HSV-1 to cells, and gC deletion mutants show reduced infectivity (Herold *et al.*, 1991). It has been observed that both mES and hES cells have different glycosylation profiles compared with differentiated cells derived from them (Atwood *et al.*, 2008; Satomaa *et al.*, 2009). It may be the case that the other HSV-1 glycoproteins are not fully matured in mES cells, thereby affecting the ability of HSV-1 to replicate to high titre in these cells.

In contrast, influenza A virus, whilst capable of entering the cells and initiating viral gene expression, failed to complete its life cycle. Viral NP, M1 and NS1 were expressed at very low levels and for a limited duration in mES cells. In addition, it has been demonstrated that changes in glycosylation of the viral haemagglutinin can hinder virus replication (Wagner *et al.*, 2000). Therefore, the unusual glycosylation profile of the mES cells may have affected haemagglutinin and neuraminidase maturation and could have been a further factor in the failure of influenza virus A/WSN/33 to produce a spreading infection. However, infection with influenza virus, even at low m.o.i., still affected the viability of mES cells.

Microarray analysis of HSV-1-infected mES cells showed that genes involved in transcription processes were upregulated following infection. This has also been observed in murine and human differentiated cell lines that are permissive for HSV-1 infection (Kamakura *et al.*, 2008; Pasieka *et al.*, 2006). In contrast, we observed that fewer genes were expressed differentially when mES cells were infected with influenza virus A/WSN/33, and genes that were enriched for transcriptional processes were downregulated. This may have been because influenza replication was restricted in these cells. Although a few genes that are part of the type I interferon response were upregulated following viral infection, there was an obvious lack in the expression of genes that are classically involved in innate immune responses to viral infection, such as nuclear factor-κB, type I interferons, interleukins and interferon regulatory factors (Geiss *et al.*, 2001; Kamakura *et al.*, 2008). This result was not unexpected, as significant upregulation of genes involved in the immune response were also not induced when hES or mES cells were infected with bacteria (Földes *et al.*, 2010; Yu *et al.*, 2009). This may be because transcription in ES cells is tightly regulated so that they remain pluripotent, and such regulation also includes interferon-stimulated genes (Szutorisz *et al.*, 2006; Yu *et al.*, 2009).

As HSV-1 can complete its full replication cycle, the potential of using high-throughput and robust mouse genetics techniques to investigate the role of host factors in virus replication becomes possible. Here, we demonstrated this utility and showed that heterozygous *Ahcyl1* KO mES cells with RNAi knockdown of the remaining expressed allele could identify host genes required to support efficient HSV-1 replication. Work by S. J. Griffiths *et al.* (unpublished data, as above) where siRNA knockdown of *AHCYL1* inhibited HSV-1 replication, and interaction between AHCYL1 and HSV-1 UL10 (gM) occurred in a yeast two-hybrid screen revealed that AHCYL1 may be a host factor involved in HSV-1 replication.

AHCYL1 is a cytosolic protein that inhibits the inositol 1,4,5-trisphosphate (IP_3_) receptor, antagonizing IP_3_-induced Ca^2+^ release from the endoplasmic reticulum, as well as regulating intracellular pH by interacting with Na^+^/$\text{HCO}{}_{\text{3}}^{-}$ co-transporters (Devogelaere *et al.*, 2008). IP_3_-induced calcium release is required for HSV-1 infection (Cheshenko *et al.*, 2003, 2007). Although the mechanism of how AHCYL1 supports HSV-1 replication is unknown, it seems that control of Ca^2+^ signalling is required to facilitate HSV-1 replication in different cell types. The generation of an *Ahcyl1* KO mouse from *Ahcyl1^+/−^* mES cells will be important for further characterization of this gene.

We showed here that mES cells may be used to investigate host–virus interactions, but we suggest that an initial detailed characterization of mES permissivity for replication of the virus of interest is essential before this system is used. If the cells are not fully replication permissive, they are still of value as they can be used to identify host factors involved in the early stages of virus replication, similar to previous work using *Drosophila* cells in an influenza screen (Hao *et al.*, 2008). Importantly, the use of *Blm*-deficient mES cells for the generation of random homozygous mutant libraries is a potentially powerful means of identifying VRDFs for viruses that infect mES cells (Li *et al.*, 2010; Wang & Bradley, 2007). However, although mES cells may be useful for identifying VRDFs or VRFs involved in the mechanics of replication, due to the lack of an innate immune response to viral infection they are not appropriate for determining host factors that influence replication through innate immunological mechanisms.

## Methods

### 

#### Cell culture.

All cell lines were cultured in a humidified atmosphere with 5 % CO_2_ at 37 °C. MDCK cells were obtained from the ATCC and cultured in Dulbecco’s modified Eagle’s medium with glutaMAX (DMEM; Gibco) with 10 % FBS (Biosera), 100 U penicillin ml^−1^ (Gibco) and 100 µg streptomycin ml^−1^ (Gibco). BHK-21 cells were also grown in this medium supplemented with 10 % tryptose phosphate broth solution (Sigma-Aldrich). The JM8 mES cells used were derived from the Agouti C57BL/6 mouse strain, substrain N. Cells were cultured in KO DMEM (Invitrogen) supplemented with 10 % FBS (Invitrogen), 2 mM l-glutamine (Invitrogen), 0.1 mM β-mercaptoethanol (Sigma) and 1000 U leukocyte inhibitory factor (Chemicon) ml^−1^ (M10 medium). JM8 mES cells that had been gene targeted in a critical exon for *Ahcyl1* to generate a heterozygous KO cell line were cultured in M10 medium supplemented with 100 µg G418 (Gibco) ml^−1^. SNLP 76/7-4 cells were cultured in KO DMEM supplemented with 7 % FBS (Invitrogen), 2 mM l-glutamine (Invitrogen) and 0.1 mM β-mercaptoethanol (Sigma). Prior to seeding mES or feeder cells, flasks or plates were coated with 0.1 % gelatine (type B; Sigma) in PBS for 10 min.

#### Viruses and infection.

HSV-1 C12, a variant that has a CMV IE1 promoter–EGFP cassette inserted at the US5 gene locus from pEGFP-C1 (Clontech), was used for these experiments (Arthur *et al.*, 2001). Virus stocks were propagated and assayed on confluent BHK-21 cells. Influenza virus A/WSN/33, a human virus that was mouse adapted in the 1940s (Hoffmann *et al.*, 2011; Ward, 1996), was also used. Virus stocks were propagated in embryonated chicken eggs and assayed on confluent MDCK cells, as described previously (Hutchinson *et al.*, 2008).

For infection experiments, cell monolayers were overlaid with a minimal volume of medium containing the appropriate m.o.i. of virus. After 1 h incubation, the inoculum was discarded and replaced with a suitable volume of culture medium. For some infections, virus was added directly to the cell suspensions and left in the culture medium, where indicated. At defined time points p.i., samples of cell-culture medium or infected cells were collected. Infected cells were lysed by three freeze–thaw cycles to release virus prior to assays. For drug inhibition, 44.4 µM ACV or 0.2 µM FIAU was added to the culture medium after the HSV-1 adsorption period.

#### Plaque assays.

Material to be assayed was serially diluted in serum-free DMEM and used to infect either BHK-21 cells for HSV-1 or MDCK cells for influenza virus WSN/33 in 12-well plates. After 1 h incubation, the inoculum was removed and the cells overlaid with DMEM containing either 5 % FBS and 0.6 % carboxymethylcellulose (Sigma-Aldrich) for HSV-1, or 0.2 % BSA (Sigma-Aldrich), 1.25 % avicel (FMC Biopolymer) and 1 µg trypsin ml^−1^ treated with l-(tosylamido-2-phenyl) ethyl chloromethyl ketone (Worthington Biochemical Corp.) for A/WSN/33 (Matrosovich *et al.*, 2006). After 2–3 days, the overlay was removed and the cells fixed with 4 % formaldehyde solution for 20 min before being stained with 0.1 % toluidine blue solution (Sigma-Aldrich) so that the number of p.f.u. could be calculated.

#### Flow cytometry.

All flow cytometric analysis was carried out using a FACScalibur (Becton Dickinson). A minimum of 10 000 gated events was acquired and analysed using CellQuest software (BD Biosciences). Antibodies were supplied by Abcam unless stated otherwise. HSV-1-infected cells were fixed in 3 % formaldehyde and detected by expression of GFP. For SSEA-1 surface staining, cells were incubated with SSEA-1 conjugated to phycoerythrin (BD Biosciences) diluted 1 : 10 in staining buffer (1 % FBS in PBS) for 1 h at 4 °C in the dark. Cells were washed twice with staining buffer and fixed for 20 min at 4 °C in 4 % paraformaldehyde (USB Corp.), followed by a further two washes with staining buffer. Intracellular staining was used for detection of influenza NP expression in influenza virus-infected mES cells. A Cytofix/Cytoperm kit (BD Biosciences) was used following the manufacturer’s instructions, with the cells being incubated with anti-NP antibody (diluted 1 : 500), followed by incubation with a goat anti-mouse IgG secondary antibody conjugated to DyLight 488 (1 : 100).

#### Confocal microscopy.

JM8A1.N3 cells were grown on gelatine-coated coverslips in a 12-well plate (2.5×10^5^ cells per well) and infected with HSV-1 at an m.o.i. of 0.1. The cells were fixed with 2 % paraformaldehyde for 30 min with continuous shaking. After washing twice with PBS, the cells were permeablized with 0.2 % Triton X-100 (Sigma-Aldrich) for 10 min at room temperature. Another washing step was performed with PBS containing 0.5 % BSA (Gibco) before the cells were immunostained with mAbs specific for one of the IE (ICP27, 1 : 1000), E (ICP8, 1 : 1000) or L (gC, 1 : 25 600) proteins of HSV-1 for 1–2 h on a shaker at room temperature. The cells were washed with PBS containing 5 % BSA and then incubated with a goat anti-mouse IgG secondary antibody conjugated to DyLight 549 (1 : 2000). After another washing step, the coverslips were mounted with ProLong Gold containing DAPI (Invitrogen) onto microscope slides. Analysis was performed using a LSM 510 META confocal microscope (Zeiss).

#### Electron microscopy.

Infected tissues were fixed in 2 % paraformaldehyde with 2.5 % glutaraldehyde in 0.1 M sodium cacodylate buffer (pH 7.42) with added 0.1 % MgCl_2_ and 0.05 % CaCl_2_ at 20 °C for 15 min and then incubated on ice for 45 min. Samples were then fixed in 1 % osmium tetroxide in sodium cacodylate buffer for 1 h at room temperature, mordanted with 1 % tannic acid for 30 min, stained en bloc with 2 % uranyl at the 30 % ethanol stage during dehydration and embedded in Agar 100 resin. Ultrathin sections (60 nm) were cut on a Leica UC6 ultramicrotome, contrast-stained with uranyl actetate and lead citrate and imaged on a 120 kV FEI Spirit Biotwin with a Tietz F4.15 CCD camera.

#### Western blotting.

Cells were lysed with RIPA buffer (Sigma-Aldrich) containing a protease inhibitor cocktail (Thermo Scientific) for 5 min on ice. The cell debris was removed by performing high-speed centrifugation for 10 min at 17949 ***g*** at 4 °C. Total protein was quantified using a BCA assay (Thermo Scientific) and normalized before use. Samples were mixed with 2× protein loading buffer (National Diagnostics) and heated for 10 min at 95 °C. The proteins were separated by SDS-PAGE (4–15 % Mini-PROTEAN TGX Precast Gel; Bio-Rad) under denaturing conditions and transferred to nitrocellulose membranes by wet blotting at 100 V for 1 h. The membrane was blocked with PBS with 0.05 % Tween 20 containing 5 % skimmed milk powder (blocking buffer) for at least 1 h at room temperature or overnight at 4 °C. Primary antibodies were diluted in blocking buffer and incubated with the membranes on a shaker for 1 h at room temperature. HSV-1-specific mAbs against the following proteins were used: ICP27 (1 : 1000), ICP4 (1 : 10 000), ICP8 (1 : 1000), gB (1 : 12 800) and gC (1 : 3200). Influenza-specific mAbs against NP (1 : 1000) and M1 (1 : 1000) and polyclonal rabbit antisera specific to NS1 (1 : 500) (Carrasco *et al.*, 2004) were used. Antibodies specific for the host protein AHCYL1 (1 : 1000) were used to confirm levels of expression in mES cells. An antibody specific for β-actin (1 : 1000) was used as a loading control. Membranes were washed three times with PBS containing 0.05 % Tween 20 before being incubated for 1 h with secondary antibody conjugated to HRP diluted in blocking buffer (goat anti-mouse–HRP, 1 : 4000, Southern Biotech; or swine anti-rabbit–HRP, 1 : 3000, DakoCytomation). After further washing steps, ECL Plus Western blotting detection reagent (GE Healthcare) was added to the membranes and they were then exposed to high-performance autoradiography film (GE Healthcare).

#### Expression microarray analysis.

mES cells were grown in six-well plates and mock-infected (M10 medium only) or infected with HSV-1 (m.o.i. of 5) or influenza virus A/WSN/33 (m.o.i. of 2). At 6 h p.i., RNA was extracted from the cells using an RNeasy Mini Plus kit (Qiagen) and normalized to equal mass across all samples. Probe labelling and hybridization were performed using an Illumina *Mus musculus* 6v2 microarray following the manufacturer’s instructions. The array probe summaries were calculated in BeadStudio (Illumina) and quantile normalized. Data were analysed using MultiExperiment Viewer (TM4 Microarray Software Suite; Saeed *et al.*, 2003, 2006), applying a variance filter with a sd cut-off of 0.1. Functional analysis of genes that were expressed differentially was performed using the ontology tool Database for Annotation, Visualization and Integrated Discovery (david) (Huang *et al.*, 2008, 2009). Significantly upregulated genes were also compared with the interferome database (Samarajiwa *et al.*, 2009). The microarray data are publicly available in ArrayExpress under accession numbers E-MTAB-882 and E-MTAB-883.

#### Cell viability.

For determination of cell viability, ATP from metabolically active cells was measured using a CellTiter-Glo luminescent assay (Promega) following the manufacturer’s instructions. Briefly, 1×10^4^ mES cells per well in 50 µl M10 medium were seeded in gelatinized, white opaque 96-well plates. An additional 50 µl M10 medium containing virus was added to the plates as follows. Cells were mock-infected (M10 only) or infected with HSV-1 at m.o.i. of 0.5, 1 or 5 or with influenza virus A/WSN/33 at m.o.i. of 0.01 or 0.1. Infections were carried out in triplicate. After 24 or 48 h incubation at 37 °C and 5 % CO_2_, 100 µl CellTiter-Glo reagent per well was added. After 2 min on a shaker followed by 10 min incubation at room temperature, the luminescence was recorded using a FLUOstar Omega (BMG Labtech) with an integration time of 0.24 s per well. The luminescence signals of virus-infected cells were compared with those of mock-infected cells to determine the effect of infection on cell viability.

#### RNAi knockdown in mES cells.

RNAi transfection mixes were set up according to the manufacturer’s instructions. Briefly, 3 µl Lipofectamine RNAiMAX (Invitrogen) was combined with ON-TARGET plus SMART pool mouse *Ahcyl1* or non-targeting siRNAs (Thermo Scientific) at a final concentration of 50 nM in opti-MEM (Invitrogen) for 20 min at room temperature. Wild-type (JM8A1.N3) or heterozygous KO *Ahcyl1^+/−^* mES cells (EPD0336_2_C12 Ahcyl1^tm1a(EUCOMM)Wtsi^; Skarnes *et al.*, 2011) were plated at 3×10^5^ per well in gelatine-coated 12-well plates followed by the addition of transfection mixes. The medium was changed daily, with a sample of cells being harvested at 48 h post-transfection to confirm gene knockdown by Western blotting. At 48 h post-transfection, cells were also infected with HSV-1 at an m.o.i. of 0.5. Samples of cell lysates were collected at 24 h p.i. and the viral titre was determined by plaque assays.

#### RNAi knockdown in HeLa cells.

siRNA SMARTpools at 0.3 µM were dispensed in 10 µl volumes using a Rapidplate384 liquid handler (Qiagen) into triplicate black 384-well plates (Corning), sealed with adhesive seals (ThermoFisher) and plate lids, and stored at −80 °C until needed (minimum 24 h, maximum 48 h). On the day of transfection, assay plates were thawed at room temperature and 10 µl transfection reagent (Dharmafect 1; Dharmacon), diluted in Hanks’ buffered saline solution (ThermoFisher) to give a final concentration of 0.1 %, was added using a Multidrop 384 (ThermoFisher). Plates were incubated for 20 min at room temperature to allow formation of transfection complexes. During complex formation, low-passage (passage 20–22) HeLa cells (ECACC) from ~50 % confluent flasks were washed in PBS and trypsinized in trypsin/EDTA (Lonza) before diluting in phenol red-free, antibiotic-free transfection medium [DMEM/F-12 (1 : 1) with 5 % FCS with 15 mM HEPES and l-glutamine; Gibco]. Using the Multidrop 384, 3×10^3^ cells in 40 µl medium were added to each well. Plates were incubated for 48 h at 37 °C and 5 % CO_2_ before infection. For infection, the medium was removed from the plates by inversion, and 10 µl medium only or HSV-1 (m.o.i. of 0.5) was added using the Multidrop 384. Plates were incubated at 37 °C for 1 h before 50 µl medium was added and the plates returned to the incubator prior to monitoring virus replication. Replication was monitored as a function of EGFP fluorescence from 24 to 80 h p.i. using a POLARstar OPTIMA plate reader (BMG Labtech). Virus replication slopes over the linear phase were calculated and normalized to transfected wells on individual assay plates, and the mean replication slope from six replicates was used for subsequent data analyses.

#### qPCR for siRNA knockdown in HeLa cells.

HeLa cells were mock-transfected or transfected with *AHCYL1* SMARTpool siRNAs in 96-well plates, in triplicate, as described above. After 48 h transfection, the medium was removed and the cells rinsed in PBS and lysed in 100 µl TRIzol (Invitrogen). Triplicate wells were combined and RNA was extracted by standard phenol/chloroform extraction methods. TaqMan qPCR was used to determine mRNA levels, using a one-step RT-qPCR kit (Thermofisher), with *AHCYL1*-specific primers (5′-TGGTGTGTGGCTATGGTGAG-3′ and 5′-GGGGTCGATTTCGGTAATGT-3′) and probes from the Universal Probe Library (Roche). Expression levels were normalized to the housekeeping cellular gene *HPRT1* (5′-TGACCTTGATTTATTTTGCATACC-3′ and 5′-CGAGCAAGACGTTCAGTCCT-3′) and calibrated against mock-transfected cells. qPCR was carried out in duplicate for each sample, and the mean level of normalized expression levels was determined.
